# Household Smoke-Exposure Risks Associated with Cooking Fuels and Cooking Places in Tanzania: A Cross-Sectional Analysis of Demographic and Health Survey Data

**DOI:** 10.3390/ijerph18052534

**Published:** 2021-03-04

**Authors:** Mazbahul G Ahamad, Fahian Tanin, Nawaraj Shrestha

**Affiliations:** 1 University of Nebraska–Lincoln, Lincoln, NE 68583, USA; nawaraj.shrestha@huskers.unl.edu; 2 Independent Researcher, Sylhet 3100, Bangladesh; fahian.tanin@gmail.com

**Keywords:** cooking place, cooking fuel, smoke producing fuel, smoke exposure risk, social determinants, indoor air quality, demographic and health survey, Tanzania, sub-Saharan Africa

## Abstract

Household smoke-exposure risk (SER) can be defined through the assessment of cooking fuels (smoke and no smoke-producing) and cooking places (indoor and outdoor) related information, which represent different levels of household air pollution. This study aimed to explore the prevalence and geographical variations in smoke-exposure risks (SERs) associated with indoor and outdoor cooking practices and use of smoke-producing and non-smoke-producing cooking fuels in Tanzania. We further investigated the social and spatial features associated with household SERs. We defined an indicator variable, the household SER, using country-level, cross-sectional data on cooking fuels and cooking places obtained from the 2015–2016 Tanzania Demographic and Health Survey and then estimated zone-level average SERs. We used an ordered logistic regression model to assess the social and spatial characteristics associated with household SER. We identified 76.4% of the 12,425 households that practiced indoor cooking using smoke-producing fuels as having a high SER. High-level SER was more prevalent in the Central, Southern highland, and Southwest highland zones. Overall, wealthier households, female-headed households, and households with higher education attainments were more likely to be categorized as households with very low SER. Meanwhile, households headed by older individuals and with larger family sizes were less likely to be in the very low SER category. The prevalence of high SER is a major public health concern in Tanzania. Improved cooking stoves and cleaner fuels should be adopted simultaneously to minimize the adverse effects associated with household SER.

## 1. Introduction

Globally, household air pollution is a major health risk. Annually, 3.8 million people are estimated to die prematurely from illnesses associated with smoke exposure and the effects of indoor air pollution produced by unclean cooking fuels [1]. In sub-Saharan African countries, the prevalence of household air pollution is very high, attributed to a dependence on cheaply available biomass and solid cooking fuels (e.g., wood and charcoal) [2,3], with a high risk of smoke exposure. In Tanzania, burning biomass and solid fuels has been associated with both indoor and outdoor air pollution and identified as the fourth leading cause of various air pollution-related diseases [4,5].

In both rural and urban Tanzania, the primary sources of cooking fuels in most households are wood (approximately 66%) and charcoal (approximately 27%) because of their high availability and low cost [6]. Only approximately 3% of households use bottled gas [6]. Electricity use among rural households is also very low, at less than 2% [7]. Because the majority of households use smoke-producing cooking fuels, roughly 97% of households (on average, 67% of rural and 30% of urban households) are exposed to high levels of smoke produced by indoor cooking. Several non-communicable diseases, including stroke, heart disease, and lung cancer, have been associated with high levels of household air pollution, which represents a top risk factor for morbidity and mortality in Tanzania [8]. In Tanzania, approximately 60 deaths per 100,000 people can be attributed to household air pollution [9].

Sociodemographic, economic, and spatial factors have been linked to the risk of household air pollution and the consequent health impacts associated with the use of cooking fuels in sub-Saharan African countries. Various factors, such as age, gender, education, socioeconomic status, and place of residence, have been reported to be related to the effects of both indoor and outdoor smoke pollution [3,4,10,11,12,13]. As many as 33% of households in Tanzania cook inside of their houses and 94% use solid or smoke-producing cooking fuels [6], which are directly related to household smoke exposure. Therefore, understanding the prevalence, geographical variations, and factors associated with household smoke-exposure risks (SERs) attributed to the use of different types of cooking places and cooking fuels remains important. Existing studies have been limited to the examination of either cooking fuels or cooking places in rural areas, with little research examining the various levels of household SERs in combining cooking fuels and cooking places.

Understanding the prevalence of SERs, including the contributions of geographical variations and associated factors, is an important goal for public health researchers and policymakers because high SERs are associated with poor household health. Therefore, we defined four levels of household SERs, based on the type of cooking place and cooking fuel used in different households. Using cross-sectional survey data for Tanzania, we compared SER prevalence rates across both rural and urban households according to sociodemographic and survey zone characteristics. We further examined the social and spatial factors associated with the prevalence of indoor and outdoor cooking and the use of smoke- and non-smoke-producing cooking fuels in both rural and urban Tanzania.

## 2. Materials and Methods

### 2.1. Data

The present study used data from the nationally representative 2015–2016 Tanzania Demographic and Health Survey (TDHS) [6], which employed a two-stage, stratified, random sampling technique to select household samples. The TDHS conducted this survey as follows: first, a total of 608 sample points (clusters) were selected randomly, representing enumeration areas for 25 regions from the Tanzanian mainland and five regions from Zanzibar, which included 13,376 households (an average of 20 from each cluster). Second, 8929 households from 428 rural clusters and 3634 households from 180 urban clusters were selected. Finally, 12,563 interview responses from men and women aged 15–49 were recorded from 12,767 occupied households. Further details on the survey design and questionnaire can be found in Chapter 1 of the 2015–2016 TDHS report [6].

### 2.2. Determination of Household Smoke-Exposure Risks (SERs)

The outcome variable used in our study was an ordered categorical variable that represented four different levels of SERs associated with the use of different types of household cooking fuels and cooking places. First, we categorized each cooking fuel type as either a “smoke-producing fuel” or a “non-smoke-producing fuel.” Smoke-producing cooking fuels included kerosene, charcoal, wood, straw, shrubs, grass, animal dung, and others. Electricity and bottled gas were categorized as non-smoke-producing cooking fuels. The second variable represented the household’s cooking places. Households that cooked inside their houses or inside of a separate building were categorized as “indoor cooking place” households, whereas households that reported using outdoor cooking places were categorized as “outdoor cooking place” households. Households that cooked no food in the house or used “other” (e.g., neither indoor nor outdoor) cooking places were not considered in our analysis [14]. Details for both the Stata do-file and R script used for SER construction can be found in the Harvard Dataverse repository.

The combination of the type of cooking fuel and location of the cooking place produced an ordinal variable for SER, which could be categorized into four levels of subjective risk: high (indoor cooking using smoke-producing fuels), medium (outdoor cooking using smoke-producing fuels), low (indoor cooking using non-smoke-producing fuels), and very low (outdoor cooking using non-smoke-producing fuels). These subjective risk levels of SER were assigned [6] based on the knowledge that smoke-producing fuels are generally more dangerous to human health than non-smoke-producing fuels, and the assumption that the use of indoor cooking places is more harmful than the use of outdoor cooking places because of the increased potential for smoke exposure in an enclosed area.

### 2.3. Visualization of Household Smoke-Exposure Risks (SERs)

We calculated the cluster-level prevalence of average household SERs in various rural and urban survey zones in Tanzania, which represented 428 and 180 clusters, respectively. The exact locations of the surveyed households were adjusted to maintain anonymity; therefore, the analyzed household data only represented zone and cluster numbers. We calculated the average household SER for each cluster, with each cluster consisting of 20–22 households.

The respective shape files were collected from www.data.humdata.org (accessed on 10 October 2019). Each cluster was denoted as a color-coded dot on the map, representing the different average SER levels of a cluster. Each dot represents the average SER value of the cluster, with each cluster representing the average SER of 20–22 included households. We used the following color scheme to indicate the average SER values: yellow, for SER ≤ 1; green, for 1 < SER < 2; and red, for SER ≥ 2. We visualized the average rural and urban household SERs using QGIS version 3.8.3 [15].

### 2.4. Predictor Variables

We examined a set of categorical independent variables, such as the demographic characteristics of the household head, including gender and age, and the household’s highest level of educational attainment, size, livelihood status (i.e., wealth index), and geographical location (i.e., survey zone), combined with the total household health expenditure data. Subsequently, we clarified the associations between these variables and the identified SER levels in the regression model.

Participants’ responses regarding their current health expenditures were included in the analysis. The demographic data for household heads were categorized according to gender stratified by age as follows: 15–34 years, 35–54 years, 55–74 years, and 75–94 years. The household’s highest level of educational attainment was defined as no education, incomplete primary, complete primary but incomplete secondary, complete secondary, and higher than secondary. Family size was grouped into four broad categories according to the number of household members: one member, two to five members, six to ten members, and eleven to 49 members. Each household’s livelihood status was represented as one of five wealth quintiles: poorest, poor, medium, richer, and richest, according to Tanzania’s wealth index category [16]. In general, the wealth index is considered to serve as a proxy for a household’s livelihood status and is noncomparable to the wealth index values of other countries. Geographical characteristics were represented by the nine survey zones (i.e., Western, Northern, Central, Southern highlands, Southern, Southwest highlands, Lake, Eastern, and Zanzibar) and the two residence area types (i.e., rural and urban).

### 2.5. Statistical Analysis

Descriptive statistics of the outcome and predictor variables were calculated using the recommended sample weight, presented as unweighted numbers and weighted percentages. We then regressed the household SER onto each household’s social and spatial characteristics. Given the association of different sociodemographic and economic characteristics with a household’s selection of cooking places and fuels, we included previously reported predictor variables (i.e., health expenditures, gender of the household head, age of the household head, household’s highest level of educational attainment, family size, wealth index, and survey zone). We also included a zone-level variable to predict the associations between SER and regional attributes. Ordered logistic regression models were used to estimate the odds ratios (ORs) and 95% confidence intervals (95% CIs) for the associations of various factors with household SER in Tanzania for the three models, including one model focused on combined rural and urban data to provide national-level associations and two models that separately focused on rural and urban characteristics. All descriptive and statistical analyses were performed in Stata version 16.1 [17]. The DHS Program of ICF International, Fairfax, VA, USA, approved the data access for the study. Institutional review board approval was not sought for this analysis because we used de-identified and publicly available 2015–2016 TDHS data for analyses.

## 3. Results

### 3.1. Prevalence of Household Smoke-Exposure Risks (SERs)

Among the 12,425 surveyed rural and urban households, 76.4% (95% CI: 74.56–78.12%) of households reported the regular practice of indoor cooking with smoke-producing cooking fuels, indicating a high overall SER among the present study sample (Table 1). Medium- and low-SER households represented 20.2% (95% CI: 18.68–21.77%) and 3.3% (95% CI: 2.38–4.52%) of the total survey population, respectively. Only 0.1% (95% CI: 0.00–0.25%) of households reported cooking outside using non-smoke-producing fuels and were categorized as very low SER.

### 3.2. Geographical Variations in Household Smoke-Exposure Risks (SERs)

**Figure 1:**
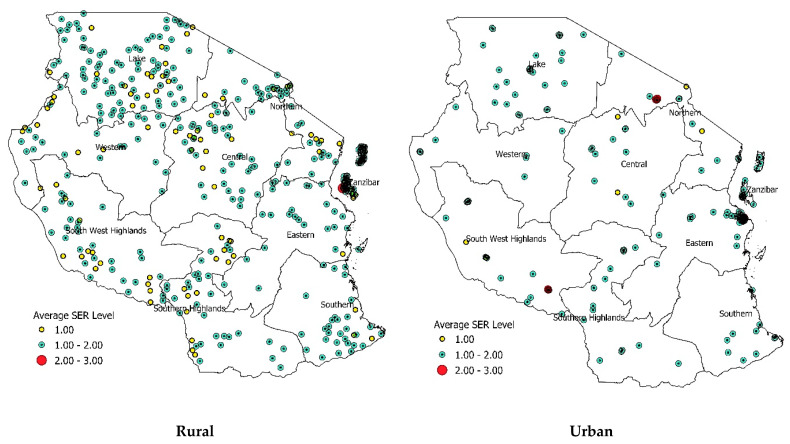
Cluster–level prevalence of average household smoke-exposure risk by survey zone in Tanzania. Each dot represents the average household smoke-exposure Risk (SER) value of the cluster, with each cluster representing the average SER of 20–22 included households. Please refer to the Section 2.2 and Section 2.3. for details.

We also identified geographical variations in household SER prevalence. High-SER households were more prevalent in rural areas (84.3%) than in urban areas (60.1%). Meanwhile, medium- and low-SER households were more prevalent in urban areas (30.6% and 8.8%, respectively) than in rural areas (15.1% and 0.6%). Figure 1 depicts the rural–urban heterogeneity in the prevalence of different household SER levels. Although the patterns of variation in SER prevalence rates across zones were more or less similar between rural and urban areas, both rural and urban areas located in the Central zone included high-SER households. Zanzibar showed relatively little variation between rural and urban households. Differences were more pronounced in other survey zones. Differences in the prevalence of medium, low, and very low SERs between rural and urban areas were generally very low and sometimes indistinct. The sociodemographic, economic, and spatial characteristics of the surveyed households are presented in Table 2.

### 3.3. Factors Associated with Household Smoke-Exposure Risks (SERs)

According to the regression analyses, we found that families with a female household head, higher levels of educational attainment, higher livelihood status (i.e., higher wealth quintile), and living in the Eastern zone were positively associated with the very low SER category at the national level (Table 3). The associations and magnitudes of the estimates calculated for the individual rural and urban household models were similar to those estimated using the combined household model, although the levels of significance varied. The likelihood of a female-headed household being categorized as very low SER was significantly higher compared with male-headed households (OR: 1.3, 95% CI: 1.14–1.48). Among urban households, this relation (OR: 1.4, 95% CI: 1.14–1.66) was also significant, whereas for rural households, the results were insignificant. Especially for urban areas, households with higher levels of educational attainment (OR: 3.4, 95% CI: 1.93–5.87) were more likely to be categorized as very low SER households than households with no educational attainment. Households categorized as middle (OR: 1.4, 95% CI: 1.07–1.82), richer (OR: 1.7, 95% CI: 1.28–2.18), and richest (OR: 3.3, 95% CI: 2.51–4.43) in terms of the wealth index were associated with high odds of being classified as low SER households, compared with the remaining wealth index categories combined. However, the distinction between rural and urban households was not significant for this variable. Households in the Eastern zone were associated with high odds of classification as very low SER households compared with those in the Western zone, regardless of the model used.

We also found that older household heads and larger family sizes were negatively associated with being classified as a very low SER household. Households headed by individuals aged 35 to 94 years had the lowest odds of being classified as very low SER households, according to results obtained using both the rural and urban models. In general, the odds of being classified as a very low SER household compared with all other SER categories were 0.8-fold (95% CI: 0.70–0.91) lower among households with heads aged 35 to 54 years, 0.6-fold (95% CI: 0.50–0.72) lower among households with heads aged 55 to 74 years, and 0.5-fold (95% CI: 0.38–0.71) lower among households with heads aged 75 to 94 years, compared with households with heads aged 15 to 34 years.

Similarly, households with more family members had lower odds of being categorized as a very low SER household. Overall, the odds of being categorized as a very low SER household compared against all other SER categories were 0.7-fold (95% CI: 0.56–0.83) lower among households with two to five members, 0.6-fold (95% CI: 0.44–0.69) lower among households with six to 10 members, and 0.5-fold (95% CI: 0.34–0.67) lower among households with 11 to 49 members, compared with single-member households. The estimated results for both the rural and urban models were similar to those for the national-level model, except for the level of significance.

We observed no associations between health expenditures and SER categorization in any of the three models examined. In most cases, the survey area and SER category showed no association with respect to the national, rural, or urban levels, except for the Eastern zone (Table 3). Similarly, no associations were observed between educational attainment and SER in rural/urban areas, especially for the first four categories of educational attainment (i.e., incomplete primary, complete primary, incomplete secondary, and complete secondary).

## 4. Discussion

To the best of our knowledge, this work is the first country-level study to assign different levels of household SER based on household use of indoor and outdoor cooking places and smoke-producing and non-smoke-producing cooking fuels using cross-sectional survey data. Our study found that a high percentage of households in Tanzania could be categorized as high SER households that practiced indoor cooking with smoke-producing cooking fuels. Overall, households headed by females, with higher levels of educational attainment, higher livelihood status, and living in the Eastern zone were associated with higher probabilities of being very low SER households. In contrast, having older household heads and larger family sizes were associated with low probabilities of being very low SER households. In most cases, the estimated relations modeled for rural and urban households were similar to those modeled at the national level.

In the present study, almost three-quarters of Tanzanian households were categorized as being high SER because of the use of indoor cooking practices with smoke-producing fuels, and the prevalence of these households was higher in rural than in urban areas. The primary sources of cooking fuel in Tanzania are smoke-producing and unclean biomass fuels [4], which are also commonly used in other sub-Saharan African countries [18]. Clean fuel, such as bottled gas, is often unavailable or costly; therefore, households typically choose to use cheap, readily available fuel sources, such as wood, for daily cooking [19]. In addition, 45% of Tanzanian households cooked in a separate space, whereas 45% of households continued to use their homes as cooking places. These rates were expected because 49.1% of the total population of Tanzania lives below the poverty line. Convincing these households to establish separate cooking places or adopt clean fuels, such as bottled gas, would, therefore, be extremely challenging [20]. Instead, promoting open-air cooking practices, under an overhang and without enclosed walls, when using wood or charcoal fuels represents a more feasible option that can be promoted to reduce the SER levels in households in many rural areas, although other feasible options (e.g., bioethanol) should continue to be explored. Thus, the limited availability and high costs of clean fuels likely influence the adoption of cooking practices that utilize smoke-producing fuels, increasing household SER.

The use of traditional but inefficient cooking stoves also contributes to household air pollution [21]. Seasonality can also affect adopted cooking methods. For example, outdoor cooking may be impossible during rainy or cold seasons [22,23,24]. In these cases, indoor cooking might be made less risky through the introduction of proper ventilation, which can have important implications for the reduction of household SER. Prospective research examining indoor and outdoor SERs could shed light on seasonal cooking practices, including the geographical availability of clean fuels. Cluster-level data on clean fuel availability and proximity to the nearest shops will be important to elucidate area-based cooking fuel availability and access.

Education is important for the adoption of cleaner, non-smoke-producing cooking fuels, as well as safe cooking practices. In most cases, education generates awareness regarding the potential negative health impacts of indoor smoke on overall household health [19,25]. In our study, we observed that households with a higher level of educational attainment were more likely to be very low SER households, indicating that households with high educational attainment levels were more likely to practice outdoor cooking using cleaner cooking fuels [26]. Awareness programs and short-term health education intervention initiatives might represent a policy option in Tanzania for reducing risky cooking practices and minimizing SER levels; safe cooking practices, including the use of properly ventilated cooking places and clean fuels, should be promoted [27].

We observed geographical variations in SER levels. Most of the zones surveyed had a high prevalence of high SER households. However, households in the Eastern zone were more often categorized as low-risk households. In the Eastern zone, 34.5% of households reported cooking outdoors, and 9.4% of households used non-smoke-producing fuels. The geographical disparities observed for outdoor cooking using non-smoke-producing fuels were also associated with variations in economic levels. We found that nearly 53.3% of the households in the Eastern zone were categorized in the richest household category. As previously mentioned, the use of clean fuel depends on the access to and availability of clean fuels, which may contribute to the high percentage of households that used non-smoke-producing fuels in this area. The high percentage of outdoor cooking practices in this zone was also associated with a low prevalence of low household SER.

Joint public–private initiatives, such as the “Clean Air Initiative of Sub-Saharan Africa”, “Promoting Bio-Ethanol as a Clean Alternative Fuel for Cooking in Tanzania”, and “Clean Cookstoves and Fuels Alliance of Tanzania”, are important for Tanzania’s national progress [28]. However, the risks and burdens of household smoke exposure and associated public health issues related to high levels of air pollution can be reduced through the gradual transition to efficient cooking stoves, particularly in areas characterized by low accessibility and low availability of non-smoke-producing fuels. The increased adoption of clean fuels can also be promoted through regionally prioritized multisectoral (e.g., public health, energy, and environment) development programs [21,29]. Common market and non-market barriers to clean fuel use (e.g., financing and marketing of clean fuel-related infrastructures, public awareness, and willingness to use clean fuel) must be addressed through public–private partnerships to improve adoption [30].

This study has several limitations. First, the survey only recorded primary cooking fuel use and location; therefore, households that used secondary cooking fuels and locations were not identified or considered in our study. The use of multiple cooking fuels and places might affect SER levels, and weighted categorization could represent a potential method for addressing this issue. Second, due to data limitations, we could not identify the degree of smoke exposure, which could also be associated with the time spent performing cooking activities. Seasonality (e.g., rainy and dry seasons), weather (e.g., windy day or night), and the availability of cooking fuels during different seasons could also represent combined factors that influence household SER. Collecting related data reflecting these events is essential for pursuing better-informed policy and decision-making related to promoting healthy cooking practices. Third, indoor smoking could be a potential contributor and might be a confounder of both high and low levels of smoke exposure risk, as this could produce smoke inside the home (please refer to Section 2.3 of 2015–16 TDHS) [6]. Finally, assessing the causal relations between the outcome and predictor variables was not possible because we used cross-sectional survey data. Multi-year cross-sectional data are required to identify any causal relations between SER and possible predictor variables. Future studies will also benefit from gathering additional details on cooking practices, such as the time spent on cooking, number of persons directly involved in cooking, and use of different cooking fuels simultaneously, to assess the degree of SER more thoroughly. It is also important to collect the data of toxic concentrates and fine particulate matter produced from cooking fuels to quantify and understand their likely impact on health outcomes.

## 5. Conclusions

A high percentage of households in Tanzania were categorized as high SER households that practiced indoor cooking with smoke-producing cooking fuels. High-level SER, which is a major public health concern in Tanzania, was more prevalent in the Central, Southern highland, and Southwest highland zones. The simultaneous adoption of improved cooking stoves and clean fuels is recommended to minimize the adverse effects attributed to household SER. Future program interventions that target these issues should consider both regional and sociodemographic factors that affect household smoke-exposure risk.

## Figures and Tables

**Table 1:** Prevalence of household smoke-exposure risks (SERs) in Tanzania.

Cooking Practice		Level of Household SER *	National (*n* = 12,425) % (95% CI)	Rural (*n* = 8870) % (95% CI)	Urban (*n* = 3555) % (95% CI)
Cooking Fuel	Cooking Place				
Smoke-producing	Indoor	High	76.4 (74.56–78.12)	84.3 (82.36–85.97)	60.1 (56.62–63.55)
Smoke-producing	Outdoor	Medium	20.2 (18.68–21.77)	15.1 (13.43–16.97)	30.6 (27.83–33.61)
Non-smoke-producing	Indoor	Low	3.3 (2.38–4.52)	0.6 (0.40–0.90)	8.8 (6.22–12.43)
Non-smoke-producing	Outdoor	Very low	0.1 (0.00–0.25)	0.03 (0.00–0.14)	0.4 (0.21–0.70)

*n*: number of observations; CI: confidence interval *. The level of smoke-exposure risk (SER) is a “subjective risk” based on the expected SER associated with the types of cooking fuels and cooking places used. All percentage (%) values are weighted. Households that used smoke-producing fuels were found to be more exposed to cooking smoke than those that used non-smoke-producing fuels. Similarly, exposure to cooking smoke was greater among households that cooked indoors than outdoors. Due to data unavailability on both cooking fuels and places, 138 household were excluded from the primary data (*n* = 12,563).

**Table 2:** Sociodemographic and economic characteristics by zone and household smoke-exposure risk (SER) categories in Tanzania.

Variable	Area *			SER †			
	National ‡ (*n*, %)	Rural (*n*, %)	Urban (*n*, %)	High (%)	Medium (%)	Low (%)	Very Low (%)
Gender of HH head							
Male	9500 (75.6)	6785 (76.0)	2715 (74.7)	77.1	19.5	3.3	0.1
Female	3063 (24.4)	2144 (24.0)	919 (25.3)	74.3	22.2	3.4	0.2
Age group of HH head							
15–34 years	3399 (27.1)	2177 (24.4)	1222 (33.7)	70.1	24.9	4.6	0.4
35–54 years	5637 (45.0)	3988 (44.7)	1649 (45.4)	76.5	20.3	3.2	0.1
55–74 years	2856 (22.8)	2183 (24.5)	673 (18.5)	81.7	15.6	2.6	0.1
75–94 years	652 (5.2)	565 (6.3)	87 (2.4)	86.4	13.4	0.3	0.0
Education							
No education	594 (4.7)	525 (5.9)	69 (1.9)	82.5	17.5	0.0	0.0
Incomplete primary	1564 (12.5)	1337 (15.0)	227 (6.3)	82.7	17.1	0.2	0.0
Complete primary	5448 (43.4)	4279 (47.9)	1169 (32.2)	79.3	20.0	0.7	0.1
Incomplete secondary	1917 (15.3)	1331 (14.9)	586 (16.1)	76.1	22.6	1.2	0.1
Complete secondary	2594 (20.7)	1346 (15.1)	1248 (34.3)	69.3	22.8	7.5	0.4
Higher	446 (3.6)	111 (1.2)	335 (9.2)	51.0	14.6	33.4	1.0
Family size							
1 member	1029 (8.2)	596 (6.7)	433 (11.9)	66.8	25.6	7.3	0.3
2–5 members	6633 (52.8)	4594 (51.5)	2039 (56.1)	74.8	21.4	3.6	0.2
6–10 members	4276 (34.0)	3236 (36.2)	1040 (28.6)	80.4	17.4	2.1	0.1
11–49 members	625 (5.0)	503 (5.6)	122 (3.4)	83.4	15.8	0.9	0.0
Wealth index							
Poorest	1992 (15.9)	1856 (20.8)	136 (3.7)	87.1	12.9	0.0	0.0
Poor	2288 (18.2)	2207 (24.7)	81 (2.2)	85.2	14.8	0.0	0.0
Middle	2560 (20.4)	2332 (26.1)	228 (6.3)	81.5	18.5	0.0	0.0
Richer	2973 (23.7)	1896 (21.2)	1077 (29.6)	75.7	23.7	0.6	0.0
Richest	2750 (21.9)	638 (7.2)	2112 (58.1)	57.0	28.5	14.0	0.6
Survey zone							
Western	858 (6.8)	668 (7.5)	190 (5.2)	84.9	14.1	0.9	0.1
Northern	1293 (10.3)	917 (10.3)	376 (10.4)	76.9	16.4	6.4	0.3
Central	1284 (10.2)	1036 (11.6)	248 (6.8)	88.0	11.4	0.7	0.0
Southern highlands	1255 (10.0)	911 (10.2)	344 (9.5)	85.0	13.3	1.6	0.1
Southern	833 (6.6)	632 (7.1)	201 (5.5)	73.9	24.9	1.2	0.0
Southwest highlands	1247 (9.9)	867 (9.7)	380 (10.5)	85.2	12.8	2.0	0.0
Lake	2555 (20.3)	1984 (22.2)	571 (15.7)	76.1	22.7	1.1	0.2
Eastern	1483 (11.8)	528 (5.9)	955 (26.3)	56.5	34.2	9.2	0.3
Zanzibar	1755 (14.0)	1386 (15.5)	369 (10.2)	79.0	17.1	3.6	0.4
Health expenditure							
No	10,612 (84.5)	7606 (85.2)	3006 (82.7)	76.8	19.9	3.1	0.1
Yes	1951 (15.5)	1323 (14.8)	628 (17.3)	73.9	21.6	4.3	0.3

*n*: number of observations; CI: confidence interval; HH: household. * Includes unweighted observations (*n*) and respective percentage (%) values. † Includes weighted percentage (%) values. ‡ Includes both rural and urban household data.

**Table 3:** Social and spatial predictors of household smoke-exposure risks (SERs) in Tanzania.

Variable	National * (*n* = 12,406)		Rural (*n* = 8854)		Urban (*n* = 3552)	
	OR (95% CI)	*p* Value	OR (95% CI)	*p* Value	OR (95% CI)	*p* Value
Gender of HH head						
Male	1.0 (Ref)		1.0 (Ref)		1.0 (Ref)	
Female	1.3 (1.14–1.48)	0.000	1.1 (0.95–1.36)	0.169	1.4 (1.14–1.66)	0.001
Age group of HH head						
15–34 years	1.0 (Ref)		1.0 (Ref)		1.0 (Ref)	
35–54 years	0.8 (0.70–0.92)	0.002	0.7 (0.63–0.89)	0.001	0.9 (0.73–1.08)	0.224
55–74 years	0.6 (0.50–0.72)	0.000	0.6 (0.49–0.74)	0.000	0.6 (0.45–0.83)	0.002
75–94 years	0.5 (0.38–0.71)	0.000	0.6 (0.39–0.81)	0.002	0.4 (0.21–0.80)	0.009
Education						
No education	1.0 (Ref)		1.0 (Ref)		1.0 (Ref)	
Incomplete primary	1.1 (0.77–1.44)	0.754	1.0 (0.69–1.43)	0.966	1.5 (0.74–2.91)	0.268
Complete primary	1.1 (0.79–1.46)	0.669	1.1 (0.74–1.53)	0.725	1.4 (0.72–2.57)	0.339
Incomplete secondary	1.1 (0.75–1.53)	0.702	1.0 (0.62–1.51)	0.871	1.5 (0.76–3.01)	0.242
Complete secondary	1.2 (0.84–1.71)	0.309	1 (0.64–1.43)	0.822	1.8 (0.91–3.64)	0.090
Higher	3.4 (1.93–5.87)	0.000	1.2 (0.58–2.37)	0.662	5.8 (2.69–12.72)	0.000
Family size						
1 member	1.0 (Ref)		1.0 (Ref)		1.0 (Ref)	
2–5 members	0.7 (0.56–0.83)	0.000	0.7 (0.52–0.91)	0.009	0.7 (0.52–0.91)	0.009
6–10 members	0.6 (0.44–0.69)	0.000	0.6 (0.47–0.88)	0.006	0.5 (0.34–0.67)	0.000
11–49 members	0.5 (0.34–0.67)	0.000	0.4 (0.29–0.68)	0.000	0.6 (0.35–1.06)	0.081
Wealth index						
Poorest	1.0 (Ref)		1.0 (Ref)		1.0 (Ref)	
Poor	1.1 (0.82–1.37)	0.670	1.1 (0.83–1.42)	0.532	0.8 (0.25–2.82)	0.766
Middle	1.4 (1.07–1.82)	0.014	1.3 (0.95–1.70)	0.106	2.5 (1.16–5.35)	0.020
Richer	1.7 (1.28–2.18)	0.000	1.2 (0.89–1.70)	0.209	1.8 (0.89–3.49)	0.105
Richest	3.3 (2.51–4.43)	0.000	3.4 (2.28–5.15)	0.000	2.4 (1.20–4.89)	0.014
Survey zone						
Western	1.0 (Ref)		1.0 (Ref)		1.0 (Ref)	
Northern	1.3 (0.83–1.94)	0.276	1.4 (0.78–2.35)	0.277	1.2 (0.66–2.12)	0.565
Central	0.8 (0.50–1.17)	0.213	1.0 (0.55–1.69)	0.891	0.5 (0.30–0.84)	0.009
Southern highlands	0.7 (0.48–1.15)	0.177	1.0 (0.51–1.84)	0.926	0.5 (0.33–0.78)	0.002
Southern	1.8 (1.17–2.85)	0.008	1.7 (0.91–3.09)	0.099	2.0 (1.33–3.04)	0.001
Southwest highlands	0.8 (0.48–1.30)	0.343	1.0 (0.49–1.96)	0.951	0.5 (0.27–0.93)	0.030
Lake	1.6 (1.12–2.35)	0.010	1.7 (1.05–2.85)	0.032	1.5 (0.98–2.19)	0.060
Eastern	2.5 (1.69–3.64)	0.000	3.6 (2.06–6.27)	0.000	1.6 (1.05–2.36)	0.029
Zanzibar	0.9 (0.58–1.38)	0.612	1.5 (0.84–2.62)	0.174	0.5 (0.25–0.95)	0.035
Health expenditure						
No	1.0 (Ref)		1.0 (Ref)		1.0 (Ref)	
Yes	1.1 (0.95–1.33)	0.159	1.0 (0.78–1.24)	0.897	1.3 (0.99–1.59)	0.056

*n*: number of observations; OR: odds ratio; CI: confidence interval; *p* value: probability value; HH: household; Ref: reference category. * Includes both rural and urban household data.

## Data Availability

Request for publicly available 2015–16 TDHS data should be made at the DHS Program website (https://dhsprogram.com/data/) (accessed on 9 October 2019). Details on Stata Do-file and R script regarding SER construction are available in the Harvard Dataverse repository (https://dataverse.harvard.edu/dataset.xhtml?persistentId=doi:10.7910/DVN/5J4YZZ) (accessed on 18 March 2020).
